# Late-manifestation of attention-deficit/hyperactivity disorder in older adults: an observational study

**DOI:** 10.1186/s12888-022-03978-0

**Published:** 2022-05-24

**Authors:** Hiroyuki Sasaki, Tadashi Jono, Ryuji Fukuhara, Kazuki Honda, Tomohisa Ishikawa, Shuken Boku, Minoru Takebayashi

**Affiliations:** 1 Department of Neuropsychiatry, Faculty of Life Science, Kumamoto University Hospital, 1-1-1 Honjo, Chuo-ku, Kumamoto-shi, Kumamoto, 860-8556 Japan; 2 Medical Center for Developmental Disorders, Department of Neuropsychiatry, Kumamoto University Hospital, Kumamoto, Japan; 3 Medical Center for Dementia-related Disease, Department of Neuropsychiatry, Kumamoto University Hospital, Kumamoto, Japan; 4 Institute for Clinical Research, National Hospital Organization Kure Medical Center Chugoku Cancer Center, Hiroshima, Japan

**Keywords:** Attention-deficit/hyperactivity disorder, Dementia, Early-onset Alzheimer disease, Late-manifestation, Late-onset

## Abstract

**Background:**

The age of attention-deficit/hyperactivity disorder onset is usually during the first 12 years of life; however, there have been recent reports of late-onset attention-deficit/hyperactivity disorder. These reports have been limited to that of young adults, and details in older adults remain unknown. As such, we had previously presented the first case report of “very” late-onset attention-deficit/hyperactivity disorder, wherein the symptoms presented in senile age. In this observational study, we aimed to investigate the prevalence and clinical features of such attention-deficit/hyperactivity disorders in older adults visiting our dementia clinic.

**Methods:**

Four hundred forty-six consecutive patients visiting our specialty outpatient clinic for dementia during the 2-year period from April 1, 2015 to March 31, 2017 were included in this study. First, the patients were examined for the presence or absence of dementia in our specialty outpatient clinic for dementia. Those not diagnosed with dementia were examined for the presence or absence of attention-deficit/hyperactivity disorder in our specialty outpatient clinic for developmental disorders. Finally, these patients who were diagnosed with attention-deficit/hyperactivity disorder were investigated in detail to clarify their clinical characteristics.

**Results:**

Of 446 patients (246 women and 200 men), 7 patients were finally diagnosed with attention-deficit/hyperactivity disorder. Although these 7 patients were initially suspected to have Alzheimer’s disease (considering their age, 6 of these 7 patients were suspected to have early onset Alzheimer’s disease), it was found that these symptoms were due to attention-deficit/hyperactivity disorder. These patients had four characteristics in common: (1) they were significantly younger than the complete study population; (2) they predominantly showed inattention-related symptoms; (3) they showed latent manifestation; and (4) they experienced a stressful life event before manifestation.

**Conclusions:**

Our previous case report suggested that very late-onset attention-deficit/hyperactivity disorder patients could be incorrectly diagnosed with dementia. In this observational study, 1.6% of patients who were initially suspected of having dementia were actually diagnosed with attention-deficit/hyperactivity disorder. This study also showed that the “late-onset” described in our previous report would be better described as “late-manifestation.” A clinician should consider late-manifestation of attention-deficit/hyperactivity disorder in the differential diagnosis when encountering dementia patients, especially early onset Alzheimer’s disease.

## Background

Attention-deficit/hyperactivity disorder (ADHD) is a developmental disorder characterized by symptoms such as impulsivity, inattention, and hyperactivity according to the criteria defined by Diagnostic and Statistical Manual of Mental Disorders, 5th edition (DSM-5) [1]. In recent years, many studies on ADHD in adults have been published [2–13], including some on the late-onset type of ADHD [8–13]. Some of the reports on late-onset ADHD have been attracting particular attention because they presented data challenging an accepted finding that the symptoms of ADHD should appear at < 12 years of age as established by studies conducted over the past 2 decades [14–17]. However, all reports on late-onset ADHD published to date were confined only to the period until early adulthood, thus making the occurrence of ADHD during the senile period questionable. Therefore, we have reported late-onset ADHD in an older adult as “very” late-onset ADHD [18]. The patient was president of her own company, and she run her business well. However, she gradually became inattentive and forgetful that it interfered with her work and daily life; thus, she visited our hospital with the encouragement of her family and employees, who worried that she was suffering from dementia. After various inspections, dementia was ruled out and she finally diagnosed with very late-onset ADHD. She was treated with methylphenidate and significant improvements in her symptoms were observed. Eventually she has returned to work and has been able to perform her daily activities without difficulty, which surprised her family and employees. This case incentivized us to conduct a perspective study on multiple cases of ADHD in order to understand and analyze its prevalence and detailed clinical features, especially in relation to the incorrect diagnosis of its symptoms as dementia.

## Methods

### Participants

Our department of neuropsychiatry at the Kumamoto University Hospital has two medical clinics: the specialty outpatient clinic for dementia, where neuropsychiatric specialists deal with different types of dementia, and the specialty outpatient clinic for developmental disorders, where developmental disorders specialists address developmental disorders. The present study was a collaborative effort between these two clinics. The subjects were 446 consecutive patients who presented at our specialty outpatient clinic for dementia during a 2-year period (from April 1, 2015 to March 31, 2017). These patients comprised 246 women and 200 men. All patients have been referred to our hospital by their family physicians for assessment of dementia. All procedures followed the Clinical Study Guidelines of the Ethics Committee of the Kumamoto University Hospital (Approval No. 622) and were approved by the internal review board. After receiving a complete description of all procedures for this study, all patients provided written informed consent.

### Materials

At our specialty outpatient clinic for dementia, all patients underwent routine laboratory tests (including thyroid, parathyroid, and vitamin B subtype tests, among others), neuroimaging studies (such as magnetic resonance imaging [MRI] and single-photon emission computed tomography), and standard neuropsychological examinations, and were diagnosed according to the DSM-IV-Text Revision criteria (this was the most current diagnostic criteria that was also relevant to our study’s objectives at the time) [19]. Depression and delirium were assessed using the Hamilton depression rating scale (HAM-D) and Delirium rating scale (DRS), respectively. Since the phosphorylated tau/amyloid beta-42 ratio in the cerebrospinal fluid (CSF) and the Pittsburgh compound-B positron emission tomography (PiB-PET) were not available at our institution at the time, only those patients who strongly requested for it were allowed to visit other institutions. Cognitive function was assessed using the Mini-Mental State Examination (MMSE) [20]. Although there are various theories on the MMSE cutoff values, several studies suggest that 26 is the optimal cutoff score (i.e., scores ≥26 should be considered) [21–23]. We therefore decided to use this value in our study. Meanwhile, at our specialty outpatient clinic for developmental disorder, DSM-5 was used to diagnose patients, and the Conner’s Adult ADHD Rating Scale (CAARS) [24] was used as an evaluation tool for our patients’ ADHD symptoms. Patients who exhibited a ≥ 30% improvement in the CAARS score during an 8-week or longer observational period were judged to have responded to the therapy. In both clinics, specialists trained in addressing dementia or developmental disorders used diagnostic criteria and rating scales relevant to the respective disorders to guarantee a certain level of accuracy.

### Procedures

Similar to a previous case [18], assessment of late-onset ADHD presenting as dementia requires specialized knowledge in both dementia and developmental disorders. Therefore, we decided to collaborate between each corresponding clinic. First, the specialists in the specialty outpatient clinic for dementia examined 446 consecutive patients. All the patients underwent several routine tests (described in the Materials subsection), and organic illness that had the potential to contribute to cognitive decline (e.g., brain tumor, vitamin B_12_ deficiency, and hypothyroidism) were ruled out. Further, for psychiatric illnesses, information was first compiled by interviewing both the patients and their family members at the outpatient reception stage. Next, a trained specialist examined the patient in person and screened the extracted symptoms and course of illness against the DSM-IV-Text Revision. Depression and delirium, which are especially easy mistaken for dementia in an outpatient dementia clinic, were further carefully evaluated using the HAM-D and DRS, respectively. After ruling out these diseases, we evaluated remaining patients whether they satisfied all the following 4 criteria: (1) MMSE score ≥ 26, (2) non-specific age-related or no significant findings on MRI, (3) no psychotic symptoms, and (4) no neurological abnormalities. Theoretically, patients who met all of the criteria (1)–(4) result in people who have visited our specialty outpatient clinic for dementia because of their concerns of possible dementia without apparent cognitive dysfunction, psychotic symptoms, neurologic symptoms, and imaging abnormalities. Patients who did not satisfy these criteria were continued to be followed up and evaluated by neuropsychiatric specialists. In cases where the patients met all four criteria, developmental disorder specialists participated in the examination and reviewed these patients’ medical records (which were prepared in our specialty outpatient clinic for dementia and contained information from the referring physicians as well as the examination history for general medical conditions). Since this phase pertained to screening and not diagnosis, over-triage was considered acceptable. For example, we required at least five ADHD symptoms; however, we did not need the symptoms to be present for more than 6 months, or the information before the age of 12 years. If these patients were suspected to have ADHD during the screening phase, they were evaluated directly by developmental disorder specialists who also interviewed their family member and relatives. While the previous phase was for screening, this phase was for diagnostic purposes; thus, we planned to adhere to the DSM-5 diagnostic criteria. The inspection of ADHD was made jointly by 2 specialists in developmental disorders. If their opinions are divided, we planned to consult with the third specialist in developmental disorder. When patients requested to receive therapeutic drugs, the effect of the therapy in these patients was also evaluated. At the time, the ADHD drugs approved for treatment of adult patients were restricted to two types in Japan: methylphenidate and atomoxetine. Patients who wished to receive ADHD treatment were also asked to select the drug that would be suitable for their lifestyle, after providing them the information regarding these drugs. Methylphenidate and Atomoxetine therapy had been begun with an initial dose of 18 mg and 40 mg respectively, which was later increased in consideration of the balance between adverse reactions and benefits. In cases in which the treatment was continued for at least 8 weeks, the treatment was deemed as effective when there was a ≥ 30% improvement according to CAARS. If the patient desired, the treatment was continued for more than 8 weeks. No other drugs were used during this assessment period. Finally, a follow-up survey was performed among patients diagnosed with ADHD 2–3 years after the study period. In the follow-up survey, the MMSE, CAARS, MRI, and DSM-5 diagnostic criteria were comprehensively evaluated for patients who were still visiting our clinic. However, for patients who had not visited our clinic, performing these procedures was not possible; therefore, questions regarding the progress of the patient’s symptoms or the emergence of any new symptoms were enquired over the phone. In the case of either of the two events, the patient was requested to undergo the same examination and reevaluation at the clinic. If the patient was unable to visit the clinic, the symptoms and progress were enquired as much as possible over the phone and the DSM-5 diagnostic criteria were applied.

### Statistical analyses

For the comparison of the groups of patients with ADHD and those diagnosed with EOAD, Mann–Whitney U test was used for the years of education and age and the Fisher’s exact test was used for the differences in gender. Mann–Whitney U test was used for the comparison of the age between the ADHD and non-ADHD groups. All tests were two-tailed, and the significance levels were set at *P* < 0.05. All statistical analyses were performed with SPSS 25.0 J for Windows (IBM SPSS Japan, Tokyo, Japan).

## Results

**Fig. 1:**
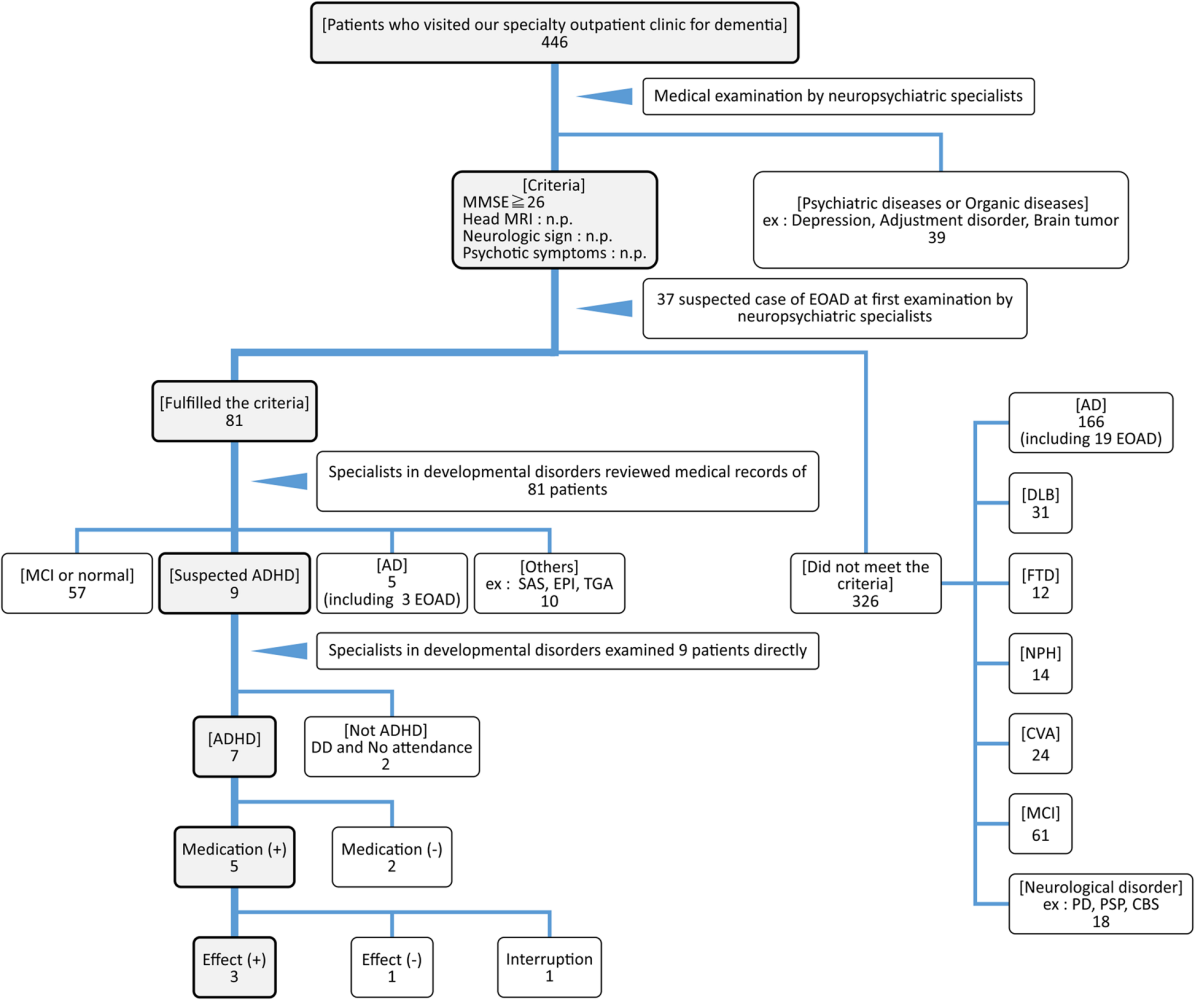
Diagnosis of ADHD in patients who visited our specialty outpatient clinic for dementia. A total of 446 patients visiting our specialty outpatient clinic for dementia were evaluated by neuropsychiatric specialists. After ruling out 39 patients with psychiatric diseases or organic diseases, 81 fulfilled our criteria and 326 did not. Developmental disorder specialists examined and reviewed the medical records for these 81 patients. As a result, 9 patients were suspected of having ADHD, and 7 of these 9 patients were given a diagnosis of ADHD. In terms of EOAD, neuropsychiatric specialists suspected 37 patients of having EOAD at the first examination. Of those 37 patients, 22 patients (3 who fulfilled our criteria and 19 who did not) were diagnosed with EOAD and 6 patients were diagnosed with ADHD. The remaining 9 patients were diagnosed with the other diseases. From the point of view of ADHD, ADHD accounted for 1.6% of patients who initially visited our specialty outpatient clinic for dementia and accounted for 16% of patients initially suspected of having EOAD

A flowchart of the diagnosis made in patients is shown in Fig. 1. First, diseases that were clearly not dementia were diagnosed in 39 patients. Of the remaining 407 patients, 326 did not fulfill our inclusion criteria and were classified as having Alzheimer disease (AD), other dementias (e.g., dementia with Lewy bodies, frontotemporal dementia), neurological diseases (e.g., progressive supranuclear palsy, Parkinson’s disease), cerebrovascular disorders, etc. Of the total number of patients, 81 patients satisfied all 4 criteria. The medical records of these 81 patients were further reviewed by the developmental disorder specialists, and 9 patients were suspected to have ADHD. Next, these 9 patients were examined directly by the specialists, and the information was also collected by interviewing their family members and relatives. One patient was diagnosed with dissociative disorder, while another patient did not re-visit the hospital, making further evaluation impossible. The remaining 7 patients were diagnosed with ADHD. Since the cases were in older adults, we were only able to interview one patient regarding ADHD symptoms before the age of 12 years (this patient was the one reported in the previous case report [18]). Therefore, the diagnosis of ADHD was made based on meeting the diagnostic criteria other than the findings before the age of 12 years. CSF and PiB-PET were requested for three patients; however, the results were negative in all the patients. After providing complete information regarding the treatment risks and benefits to the patients and their relatives, 5 patients consented to receive treatment for ADHD. Of these, 2 and 3 patients were treated with atomoxetine and methylphenidate, respectively. One patient out of these 5 patients discontinued the treatment, resulting in an insufficient period of evaluation. In another patient, the response to treatment was unclear. In the remaining 3 patients (1 and 2 patients were treated with atomoxetine and methylphenidate, respectively), the CAARS score was observed to improve by ≥30% over an 8-week period of administration, demonstrating the effectiveness of treatment with anti-ADHD drugs. In the follow-up survey, one out of 7 patients with ADHD was evaluated in detail since he was still visiting the hospital, and no progression of symptoms or new symptoms were observed since the visit. The remaining 6 patients were no longer visiting the clinic and were contacted telephonically. One patient was not heard from; however, we were able to interview 5 patients over the telephone, and no progression or new symptoms were noted.

At the time of their visit to our specialty outpatient clinic for dementia, almost all ADHD patients had been suspected of having early-stage AD, with 6 of the final 7 patients suspected of having EOAD because they were < 65 years of age. As shown in Fig. 1, neuropsychiatric specialists, at first impression, suspected 37 patients out of all 446 patients as having EOAD. Of those 37 patients, 22 patients (60%) were conclusively diagnosed with EOAD (the EOAD group) and 6 patients (16%) were diagnosed with ADHD (the ADHD group). The remaining 9 patients (24%) were diagnosed with other diseases (e.g., mild cognitive impairment and sleep apnea syndrome). That is, 1.6% of patients visiting our specialty outpatient clinic for dementia were diagnosed with ADHD, and 16% of patients in whom EOAD was initially suspected were diagnosed with ADHD.

The ADHD group included a higher number of patients with jobs indicating a high social status (e.g., company owner, university lecturer, school master, mechanical engineer, etc.) than the EOAD group. To verify this, we statistically evaluated the duration of education between the ADHD (who were initially suspected EOAD) and EOAD groups and found a significant difference (Table 1).

**Table 1:** Comparison between the ADHD and EOAD groups on the duration of education in patients

	ADHD group aged < 65 (*n* = 6)	EOAD group (*n* = 22)	P
**Educational years: median (IQR)**	15.0 (14.0–16.5)	12.0 (12.0–13.3)	0.003
**Age: median (IQR)**	55.0 (51.5–59.3)	58.5 (56.8–61.0)	0.112
**Gender: Female / Male (%)**	3 (50.0%) / 3 (50.0%)	9 (40.9%) / 13 (59.1%)	1.000

To reach a diagnosis of ADHD, each patient was individually examined, and the family members were also interviewed. This revealed four common features (Table 2) among ADHD patients.

**Table 2:** Four common features

1. The age of patients with ADHD was significantly lower than the rest of the sample	
2. The type of these ADHD cases was inattention-predominant ADHD	
3. These ADHD cases might have been a late manifestation of a long-standing disease	
4. All patients with ADHD chronically experienced significant life events, before manifestation of ADHD symptoms	

First, the age of 7 patients with ADHD (median: 56.0, quartile: 52.0–63.0) was significantly lower than the rest of the sample (median: 75.0, quartile: 69.0–81.0; *P* < 0.001). Second, while all the ADHD patients satisfied ≥5 of the inattention-related diagnostic criteria of DSM-5, none of them fulfilled the 5 criteria related to hyperactivity or impulsiveness. They inadvertently forgot things because they were otherwise preoccupied, or they forgot to perform one task while simultaneously performing several other tasks. Third, the behavioral characteristics of inattention and forgetfulness had been displayed by all these patients since they were young adults. This was revealed through information from family members and others involved, which was as follows: patients previously had characteristics similar to ADHD symptoms, although such characteristics did not affect their living activities to the level that would satisfy the diagnostic criteria of ADHD. However, their ADHD-like characteristics gradually worsened, and they began to have difficulties in their daily lives over a period ranging from a few months to a few years before the consultation, to the point that they met the diagnostic criteria for ADHD at the time of the examination. This process is considered the manifestation of symptoms that have existed for years. Fourth, several months to 1 or 2 years before ADHD began to be worse, the patients experienced significant life events, the effects of which persisted chronically, causing severe mental upheaval. The most frequent inciting event was job related (post-reshuffle, over-working, etc.), followed by issues with interpersonal relationships (discord with neighbor, problems within the family, etc.).

## Discussion

In our previous case report, we reported on late-onset ADHD with dementia-like symptoms. In this study, we discussed the possibility of several such patients presenting to the dementia outpatient clinic. We also demonstrated that it might be difficult to distinguish ADHD from other types of dementia, especially EOAD, in such patients.

There could be various reasons for these patients with ADHD to be initially suspected of having EOAD. One reason was that the age range of patients matched between the ADHD and EOAD groups (Table 1). Another reason may be the clinical course. As mentioned in Table 2 and in the Results section, all ADHD patients manifested inattentiveness and forgetfulness at the senile stage. Perhaps, this clinical course of ADHD symptoms that began to interfere with the patient’s social lives in the senile age were similar to the onset of dementia from a relative’s point of view, and this is the reason why they attended dementia clinic recommended from their surroundings. Similarly, EOAD often presents with symptoms of forgetfulness and inattention rather than behavioral and psychological symptoms of dementia, such as visual hallucinations and personality changes, and the onset of EOAD is often detected by surrounding people due to disturbed social activity of the affected patients [25]. Furthermore, patients with EOAD are known to often present with the above symptoms before the development of brain atrophy (including the hippocampus). Therefore, it is not surprising that ADHD patients in the present study who visited our specialty outpatient clinic for dementia with chief complaints of forgetfulness and inattention were initially suspected of having EOAD despite the absence of obvious morphological abnormalities.

How can ADHD be differentiated from EOAD? First, the procedures that were helpful included the PiB-PET and the determination of the phosphorylated tau/amyloid beta-42 ratio in the CSF. Both are known to have high sensitivity and high specificity. If negative results are obtained in both examinations, there is an extremely high likelihood that EOAD is absent [26]. Thus, EOAD was excluded through the use of PiB-PET and CSF, as described in our previous case report [18]. However, the problem is that these tests are special and not every hospital can do it. Therefore, in this study, there were only three patients with ADHD who could be ruled out as having EOAD even after these tests were performed. Another point is the type of forgetfulness observed in the patient. In cases of EOAD, patients become forgetful in the way that they cannot recall whether they went out the previous day, or they cannot memorize the name of the attending physician, irrespective of how many times they see the physician. However, in this study, patients with ADHD were forgetful in that they inadvertently forgot things because they were preoccupied with something else, or they forgot to perform one task while simultaneously performing several other tasks. Both types of forgetfulness were the same in terms of interfering with the patients’ living activities; however, forgetfulness in EOAD involves the lack of episodic memories, whereas that in ADHD is forgetfulness attributable to inadvertence. Given this difference, it would appear that it was easy for the specialists to distinguish between the two; however, this difference is only apparent in relatively advanced EOAD. In the case of very early EOAD, the lack of episodes is not quite obvious; hence, it remains difficult to distinguish.

In terms of treatment, 60% of the patients with ADHD responded to the treatment. In general, the main focus of dementia treatment is to prevent progression. However, in the case of ADHD, there are cases where treatment clearly improves symptoms and quality of life, as in the case shown in the previous case report. The difference in medication is one of the most important factors in differentiating between the two. Moreover, ADHD medications have also been suggested to be effective in treating diseases other than ADHD [27–29]; therefore, effective treatment does not necessarily indicate that the patient has ADHD.

Moreover, there is a persistent question that should be considered. It is now well-established that a considerable subset of people with mild cognitive impairment (MCI) remain cognitively stable over a number of years without ever progressing to dementia. It is possible that some or all of the ADHD patients constituted cases of stable MCI. This can be explained by the third finding in Table 2, which has been elaborated in detail in the Results section. Considering the clinical course of gradual worsening of ADHD-like characteristics, it is more reasonable to explain them as a manifestation of ADHD rather than as a stable MCI. However, in current practice, there may be cases in which ADHD and stable MCI cannot be distinguished with certainty, and this is an issue warranting further studies.

Previous studies on late-onset ADHD have proposed two types of late-onset ADHD; however, these studies have been limited to young adults. One type is “new-onset” ADHD [8, 9] and another type is “manifestation” of the disease [10, 12]. The result of our study corresponds to the latter. Moreover, what we described previously in our case report as “very late-onset ADHD” is actually more accurately termed as “the late-manifestation of ADHD”. Then, why did ADHD symptoms manifest in the senile age? In this regard, “aging alteration” and “stressful life events (SLE)” showed in Table 2 may play an important role. In the senile age, the brain function may have been declining due to age-related changes. In addition to these age-related changes, SLE may have accelerated decline of brain function. The relationship among SLE, late life, and cognitive decline has already been reported in another study [30]. Due to the functional brain decline caused by these factors, symptoms of ADHD may have manifested as these could not be compensated anymore. Higher intelligence quotient (IQ) of patients with ADHD has been demonstrated in previous research [9]. Our study did not compare the IQs because we were unable to apply the Wechsler Adult Intelligence Scale; however, we observed a statistically significant difference in the years of education (Table 1). Probably, some older adults with ADHD may efficiently compensate their ADHD tendency by utilizing their aptitude.

Therefore, ADHD cases in older adults can be summarized as follows. First, there are people who live with high intellectual ability and compensate ADHD well. As these people reach senile age, their brain function begins to deteriorate owing to aging alteration, and SLE further reduces their brain function. The diminished brain function is no longer able to compensate for ADHD symptoms as it used to previously. When ADHD symptoms become apparent at senile age and interfere with daily life, those around the patient may suspect development of dementia and advise the patient to see a doctor. This is probably how the patients with ADHD in this study came to visit the dementia outpatient clinic.

### Limitations

The study had certain limitations. First, there was the possibility of missed diagnosis of ADHD. In this study, all the patients underwent several routine tests, and similar to routine practice by specialists, we ruled out diseases that had the potential to contribute to cognitive decline (e.g., depression, brain tumor, vitamin B_12_ deficiency, and hypothyroidism). When these diseases were suspected, the patients were excluded from the study. However, we cannot deny the possibility that some of the excluded patients had ADHD as a comorbidity. For example, a previous study reported that older adults with ADHD often had depression [31]. Moreover, it was also problematic that the developmental disorder specialists began the intervention with verification of medical records prepared by the dementia specialists. Since impulsivity, inattention, and forgetfulness were also important symptoms in the evaluation of dementia, the dementia specialists examined these symptoms. However, since the interview was not conducted with ADHD in mind, it might not have been sufficient. Furthermore, it is possible that the medical records were inadequately documented and therefore, patients with ADHD were not considered by the specialists in developmental disorder. Therefore, a developmental disorder specialist could ideally directly examine all the outpatients and evaluate them for the presence of ADHD. However, this was not practical in routine clinical practice, neither in terms of patient consent nor in terms of manpower on the part of the researcher. Therefore, it was necessary to narrow down the list of patients to some extent by screening the medical records. The next limitation is that sufficient information before the age of 12 was not obtained because the patients were old and fewer people knew about their childhood. Unlike the screening phase, the policy during the diagnosis phase was to accurately adhere to the diagnostic criteria. However, owing to the aforementioned reasons, it was difficult to ascertain the characteristics before the age of 12 years; hence, we had to exclude this item from the diagnosis. The third limitation pertains to the diagnosis performed by two developmental disorder specialists. During the direct examination of the patients themselves or the information provided by their family performed by a developmental disorder specialist, each patient was not separately diagnosed by two specialists. In practice, the patient had first been diagnosed by a specialist, and then the result was discussed between the first specialist and a second specialist. Moreover, both specialists participated in the study with knowledge of the study’s aims. These features may have created a bias in diagnosis. The fourth limitation was about the follow-up survey. The follow-up survey permitted detailed verification of only the patient who continued to visit the clinic; however, patients who had not visited the clinic were managed over the telephone. Many details cannot be confirmed over the phone and certain findings could be missed.

## Conclusion

Our previous case report suggested that very late-onset ADHD patients could be incorrectly diagnosed with dementia. In this study, 1.6% of patients who were suspected of having dementia and who visited our outpatient clinic were actually patients with ADHD, and it was difficult to distinguish these patients from those with dementia, particularly EOAD. This study also showed that the “very late-onset” described in our previous case report would be better described as “late-manifestation”. Some reports indicate that the frequency of early-onset dementia in young patients visiting specialist clinics ranges from 7.3 to 44% [32–35]. While diagnosing these types of dementias, especially EOAD, we ought to consider older adults with ADHD as a differential diagnosis, although such consideration is ill-afforded as diagnostic criteria for dementia at this point in time. In future, we need to establish an efficient way to distinguish older adults with ADHD from dementia and follow this up on a long-term basis.

## Data Availability

The datasets generated during this study are not publicly available due to the patients’ privacy but are available from the corresponding author on reasonable request.

## Abbreviations

AD: Alzheimer disease
ADHD: Attention-deficit/hyperactivity disorder
CAARS: Conner’s Adult ADHD Rating Scale
CBS: Corticobasal syndrome
CSF: Cerebrospinal fluid
CVA: Cerebral vascular accident
DD: Dissociative Disorder
DLB: Dementia with Lewy bodies
DRS: Delirium rating scale
DSM: Diagnostic and Statistical Manual of Mental Disorders
EOAD: Early-onset Alzheimer disease
EPI: Epilepsy
FTD: Frontotemporal dementia
HAM-D: Hamilton depression rating scale
MRI: Magnetic resonance imaging
IQ: Intelligence quotient
IQR: Interquartile Range
MCI: Mild cognitive impairment
MMSE: Mini-Mental State Examination
n.p.: Not particular
NPH: Normal pressure hydrocephalus
PD: Parkinson’s disease
PiB-PET: Pittsburgh compound B positron emission tomography
PSP: Progressive supranuclear palsy
SAS: Sleep apnea syndrome
SLE: Stressful life events
TGA: Transient global amnesia
